# Insecticide-Treated Nets for the Prevention of Malaria in Pregnancy: A Systematic Review of Randomised Controlled Trials

**DOI:** 10.1371/journal.pmed.0040107

**Published:** 2007-03-27

**Authors:** Carol Gamble, Paul J Ekwaru, Paul Garner, Feiko O. ter Kuile

**Affiliations:** 1 Centre for Medical Statistics and Health Evaluation, University of Liverpool, Liverpool, United Kingdom; 2 Clinical Epidemiology Unit, Makerere Medical School, Makarere University, Kampala, Uganda; 3 Liverpool School of Tropical Medicine, Liverpool, United Kingdom; 4 Malaria Branch, Division of Parasitic Diseases, United States Centers for Disease Control and Prevention, Atlanta, Georgia, United States of America; Royal Melbourne Hospital, Australia

## Abstract

**Background:**

Protection from malaria with insecticide-treated bednets (ITNs) during pregnancy is widely advocated, but evidence of benefit has been inconsistent. We undertook a systematic review of randomised trials.

**Methods and Findings:**

Three cluster-randomised and two individually randomised trials met the inclusion criteria; four from Africa (*n =* 6,418) and one from Thailand (*n =* 223). In Africa, ITNs compared to no nets increased mean birth weight by 55 g (95% confidence interval [CI] 21–88), reduced low birth weight by 23% (relative risk [RR] 0.77, 95% CI 0.61–0.98), and reduced miscarriages/stillbirths by 33% (RR 0.67, 0.47–0.97) in the first few pregnancies. Placental parasitaemia was reduced by 23% in all gravidae (RR 0.77, 0.66–0.90). The effects were apparent in the cluster-randomised trials and the one individually randomised trial in Africa. The trial in Thailand, which randomised individuals to ITNs or untreated nets, showed reductions in anaemia and fetal loss in all gravidae, but not reductions in clinical malaria or low birth weight.

**Conclusions:**

ITNs used throughout pregnancy or from mid-pregnancy onwards have a beneficial impact on pregnancy outcome in malaria-endemic Africa in the first few pregnancies. The potential impact of ITNs in pregnant women and their newborns in malaria regions outside Africa requires further research.

## Abstract 2

A systematic review of the evidence from trials shows that use of insecticide-treated mosquito nets has a beneficial impact on pregnancy outcome in malaria-endemic Africa in the first few pregnancies.

## Editors' Summary

**Background.:**

Malaria is one of the world's most important killer diseases. It is responsible for around a million deaths every year, most of them in Africa and most of them in children. Pregnant women and their unborn babies are also at high risk. Women who have malaria become extremely weak because of anemia, they are more likely to miscarry, and their babies have low birth weights. (Birth weights under 2.5 kilograms are considered to be low; low-birth-weight babies face higher risks of sickness and death than other babies.) It has been estimated that, every year, malaria during pregnancy is responsible for the deaths of about 100,000 to 200,000 babies within their first year of life.

The parasite that causes malaria is carried by certain species of mosquito. In the areas where these mosquitoes are found, taking steps to reduce the chance of being bitten can reduce one's chances of getting infected with malaria. In Africa, these mosquitoes bite mainly in the hours of darkness, so sleeping under a mosquito net helps. During the last 20 years it has been established that mosquito nets impregnated with an insecticide provide much better protection than ordinary nets. Research has shown that children who sleep under these insecticide-treated nets (ITNs) are much less likely to get malaria than other children living in the same area. The correct use of ITNs is now being heavily promoted in many national and international programs.

**Why Was This Study Done?:**

It seems logical that pregnant women should be encouraged to sleep under ITNs, and this is recommended by the World Health Organization and other authorities. However, we cannot be sure that there are benefits, as there is a lack of clear evidence to show whether women who sleep under ITNs actually suffer less from malaria and anemia, and what the implications are for their babies.

**What Did the Researchers Do and Find?:**

They did no new work in the field or in the laboratory. Instead, they conducted what is known as a systematic review. Working to a set of criteria carefully formulated in advance, they searched the medical literature for well-conducted “randomized controlled trials” (RCTs) involving the use of ITNs by pregnant women. RCTs are studies where one group of people receives the “treatment” under investigation and another group does not. It is decided at random who goes into which group. The authors found five trials in Africa, four of which fulfilled the entry criteria for their analysis. In three of the four studies conducted in Africa, whole villages had been “randomized” to determine which pregnant women received ITNs. In another African trial, individual women had been randomized. In total, over 6,000 women were involved in the African trials. One trial had been done outside Africa, in Thailand, where just over 400 individual women had been randomized.

In the research done in Africa, it had been found that women who slept under ITNs had lower numbers of parasites in their blood. Miscarriages were much reduced—by a third in those women who were in their first few pregnancies. The overall proportion of babies who were low birth weight went down by nearly a quarter. In the research done in Thailand, the women using ITNs were less anemic and the miscarriage rate was again lower, although there was no change in the low-birth-weight figures.

**What Do These Findings Mean?:**

The evidence from the three trials done in Africa strongly suggests that it is a good idea for pregnant women to sleep under ITNs. With evidence available from only one trial conducted outside Africa, it is hard to be sure at this stage whether ITNs have the same beneficial effects for pregnant women in other parts of the tropics. More research is needed there.

**Additional Information.:**

The following organizations all have useful information about malaria on their Web sites, and we have provided links to the appropriate pages below. Please access these Web sites via the online version of this summary at http://dx.doi.org/10.1371/journal.pmed.0040107.

- US Centers for Disease Control and Prevention has information specifically about malaria during pregnancy
- Roll Back Malaria has information specifically about malaria during pregnancy
- Wikipedia—a free online encyclopedia that anyone can edit
- The World Health Organization (WHO)
- The United Nations Children's Fund (UNICEF)
- MedlinePlus brings together authoritative information from the US National Library of Medicine, National Institutes of Health, and other government agencies and health-related organizations
- The Wellcome Trust

## Introduction

Approximately 50 million pregnant women are exposed to malaria each year. Pregnant women are more susceptible to malaria, placing both mother and fetus at risk of the adverse consequences [1–3]. In areas of low and unstable transmission, such as in many regions in Asia and the Americas, women do not acquire substantial antimalarial immunity, and are susceptible to episodes of acute and sometimes severe malaria, and fetal and maternal death [4]. In areas with stable malaria transmission, such as in most of sub-Saharan Africa, infection with Plasmodium falciparum in pregnancy is characterised by predominantly low-grade, sometimes sub-patent, persistent or recurrent parasitaemia. These infections frequently do not result in acute symptoms yet are a substantial cause of severe maternal anaemia [5] and of low birth weight (LBW) [3], and as such are a potential indirect cause of early infant mortality [6–8]. Because most of these infections remain asymptomatic, and therefore undetected and untreated, prevention of malaria in pregnancy is especially important in these settings.

The World Health Organization (WHO) advocates a three-pronged approach to malaria control in pregnancy that includes the use of insecticide-treated bednets (ITNs), intermittent preventive treatment (IPT), and case management (treatment) [9]. In areas of stable malaria transmission in sub-Saharan Africa, ITNs are highly effective in reducing childhood mortality and morbidity from malaria [10]. Although ITNs are promoted as a major tool in the fight against malaria in pregnancy, the available evidence about their efficacy in pregnancy has been inconsistent. In this review, we summarise the available data from randomised controlled trials that compared the effects of ITNs to no nets, or to untreated nets, on the health of pregnant women and birth outcome.

## Methods

A protocol was developed for this review [11], and the standard search strategy of the Cochrane Infectious Diseases Group was used to identify potentially relevant trials [12]. The inclusion criteria were all trials that randomised individuals (pregnant women) or clusters (community or antenatal clinics) in areas where malaria transmission occurs. Where cluster-randomised trials were identified, the methods of analysis were checked to ensure that the precision of the data extracted from the reports was correctly estimated. The authors needed to have adjusted for clustering, as ignoring the clustering provides the correct point estimate of the magnitude of the trial effect but may overestimate the precision, resulting in potentially incorrect conclusions [13]. Primary outcomes selected were mean haemoglobin and anaemia, and mean birthweight and LBW; secondary outcomes included peripheral malaria in the mother assessed by finger prick during pregnancy or at birth, placental malaria assessed by microscopy, clinical malaria, pre-term birth, fetal loss (defined as miscarriage or stillbirth), and maternal death.

Trial quality was assessed as adequate, inadequate, or unclear based on the methods used to generate the allocation sequence and allocation concealment [14]. Minimisation of loss to follow-up was considered adequate (≥90% of the participants randomised included in the analysis), inadequate (< 90%), or unclear (not reported).

Outcomes were combined using the inverse variance method in RevMan [15,16]. We used the fixed-effects model throughout, and assessed heterogeneity by the *I* ^2^ test (with values of >50% representing moderate heterogeneity) [17]. To minimise the anticipated heterogeneity, no attempt was made to combine trials that compared ITNs to no nets and those that compared ITNs to untreated nets [10]. Because all the included studies from Africa compared ITNs to no nets, and the one study comparing ITNs to untreated net was conducted in Thailand, this also resulted in stratification by the major malaria transmission regions (Africa versus non-Africa), which differ in transmission intensity, parasite species, predominant vector, and vector behaviour.

The effect of ITNs was expected to be greatest in the first few pregnancies because women develop pregnancy-specific immunity against placental parasites over successive pregnancies as a consequence of repeated exposure [18]. Because gravidity was considered the greatest potential modifier of the effect of ITNs, analyses were stratified a priori by gravidity groups whenever this was possible based on the details provided.

Other potential sources of effect modifications that were explored included concomitant use of IPT in pregnancy (IPTp), and differences between trials that used individual randomisation, in which women benefit primarily from personal protection by treated nets, and trials that used cluster randomisation. In the latter trials, ITNs were distributed to whole communities, which may result in a mass or community effect due to area-wide killing of the malaria-transmitting mosquitoes [19–21]. Women in the cluster-randomised trials were mostly provided with ITNs prior to becoming pregnant and were thus protected throughout pregnancy. In the individually randomised trials, nets were provided as part of antenatal care, i.e., typically from 20 to 24 wk onwards. We could not explore other potential sources of heterogeneity because the number of trials identified was too few.

## Results

### Description of Trials

Six trials were identified; we excluded one trial as the analysis had not adjusted for clustering, and loss to follow-up was high (Text S1) [22]. Of the five included trials (Table 1), two were individually randomised [23,24], and three were cluster-randomised with analysis that took design effects into account [25–27].

**Table 1:**
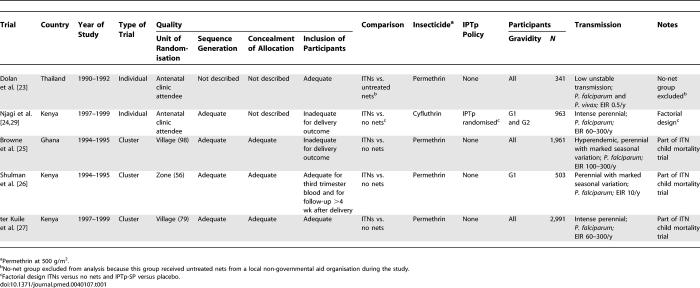
Characteristics of Included Studies

Four trials were conducted in stable malaria-endemic areas in Africa (three in Kenya [24,26,27] and one in northern Ghana [25]), all with entomological inoculation rate (EIR) > 1/y, and one in Karen refugee camps along the Thailand–Myanmar border in an area with low and markedly seasonal malaria where P. falciparum and P. vivax coexist (EIR 0.5/y) [23].

The African trials compared ITNs to no nets; 6,418 women were enrolled [24–27]. The remaining trial from Thailand randomised individual women to receive either ITNs, untreated nets, or no nets [23]. In the “no nets” arm, a large proportion of women received nets from another donor independent of the study, and the researchers split the results in this control arm into women using donor nets and women not using donor nets. Because this compromised the validity of the control arm, we included only the comparison of ITNs with untreated nets (*n =* 223).

All African trials gave double- or family-sized nets to each household. The nets used in Thailand were smaller single-sized nets (70 × 180 × 150 cm). All trials used the widely available insecticide permethrin (500 g/m^2^), except one trial that used cyfluthrin [24].

One trial included IPTp-SP in a factorial design [24]. Women were allocated to receive (1) ITNs plus IPTp-SP, (2) IPTp-SP alone, (3) ITNs plus IPTp-SP placebo, or (4) IPTp-SP placebo alone (“control”). None of the other trials included IPT.

In the four trials from Africa, only women having their first baby were included in one trial [26], women having their first or second baby in another [24], and women of all gravidity in the remaining two trials (Table 1) [25,27]. In the trials including pregnant women of all gravidity, the authors analysed them differently: ter Kuile et al. grouped by gravidity 1 to 4 (G1–G4) and gravidity 5 and above (G5+) [27]. Browne et al. grouped by first pregnancy (G1), second pregnancy (G2), and third pregnancy and above (G3+) for continuous endpoints [25]. To allow for sub-group analysis by gravidity group, we grouped the G3+ group from Browne et al. and the G5+ group from ter Kuile et al. into one sub-group, referred to as “high gravidity”, and the G1 from Shulman et al., the G1 and G2 groups from Browne et al. and the G1–G4 group from ter Kuile et al. into another sub-group, referred to as “low gravidity” [25–27]. The study by Browne et al. also provided sub-group analyses for dichotomous endpoints, but unlike in the analysis for continuous endpoints they were not adjusted for cluster randomisation [25].The study by Dolan et al. in Asia did not provide estimates by gravidity group, with the exception of the effect on birth weight [23].

### Treated Nets versus No Nets (Four Trials in Africa)

#### Primary outcomes.

**Figure 1:**
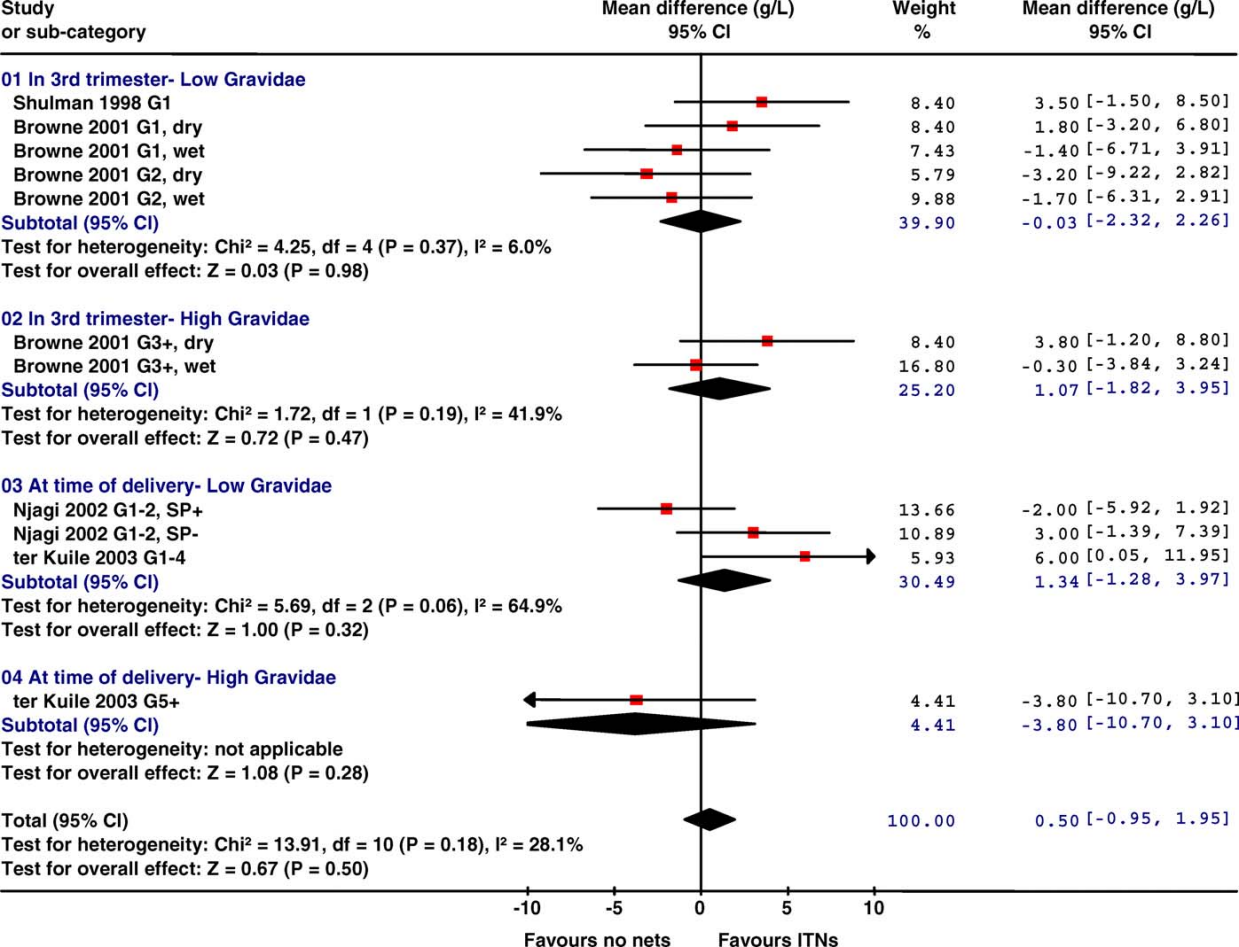
Effect of ITNs versus No Nets in Africa on Mean Haemoglobin Levels (in Grams/Litre) The red squares represent the effect estimates of ITNs; the black lines represent the 95% confidence intervals associated with the effect estimates (a line with an arrow indicates that the confidence interval was greater than could be illustrated in the graph). The black diamonds represent the summary effect estimates for the different subgroups (“subtotal”) and for the overall effect (“total”). “Dry” and “wet” refer to the dry and wet seasons. SP+, women randomized to IPTp-SP; SP-, women randomized to receive placebo ITPp (factorial design).

All four trials reported the effect of ITNs on haemoglobin (Hb) levels and anaemia. Because of the variations in trial design and reporting, it was not possible to combine the results from all four trials for anaemia (Hb < 100 or 110 g/l) and severe anaemia (Hb < 70 or 80 g/l) [28]. The results for mean haemoglobin are provided by the time of assessment (third trimester or delivery) and by gravidity group (Figure 1).

There was no evidence for improved haemoglobin levels in women having their first or second babies in the two trials that assessed haemoglobin levels in the third trimester [25,26]. The overall (i.e., all gravidae) summary odds ratio (OR) for any anaemia in the third trimester was 0.88 (95% confidence interval [CI] 0.71–1.10, *p =* 0.26, one trial) and for severe anaemia was 0.77 (0.56–1.08, *p =* 0.13, two trials). Insufficient details were reported to provide sub-group analysis by gravidity group.

There was significant heterogeneity of treatment effect between the two other trials and sub-groups that assessed haemoglobin levels at delivery, with no evidence for a consistent effect overall (Figure 1) [24,27]. Mean haemoglobin levels were significantly higher in G1–G4 in the trial by ter Kuile et al., who also reported a significant delay in the time to the first episode of any anaemia (Hb < 110 g/l) in G1–G4 (hazard ratio [HR] 0.79, 95% CI 0.65–0.96, *p =* 0.02), but not in G5+ (HR 1.00, 0.86–1.18, *p =* 0.97) [27]. Njagi et al. did not find a significant increase in the mean haemoglobin levels of primi- and secundigravidae (Figure 1) or a significant overall reduction in any anaemia, although sub-group analysis by gravidity showed that a significant reduction in any anaemia was found in primigravidae and not secundigravidae (not shown) [29].

**Figure 2:**
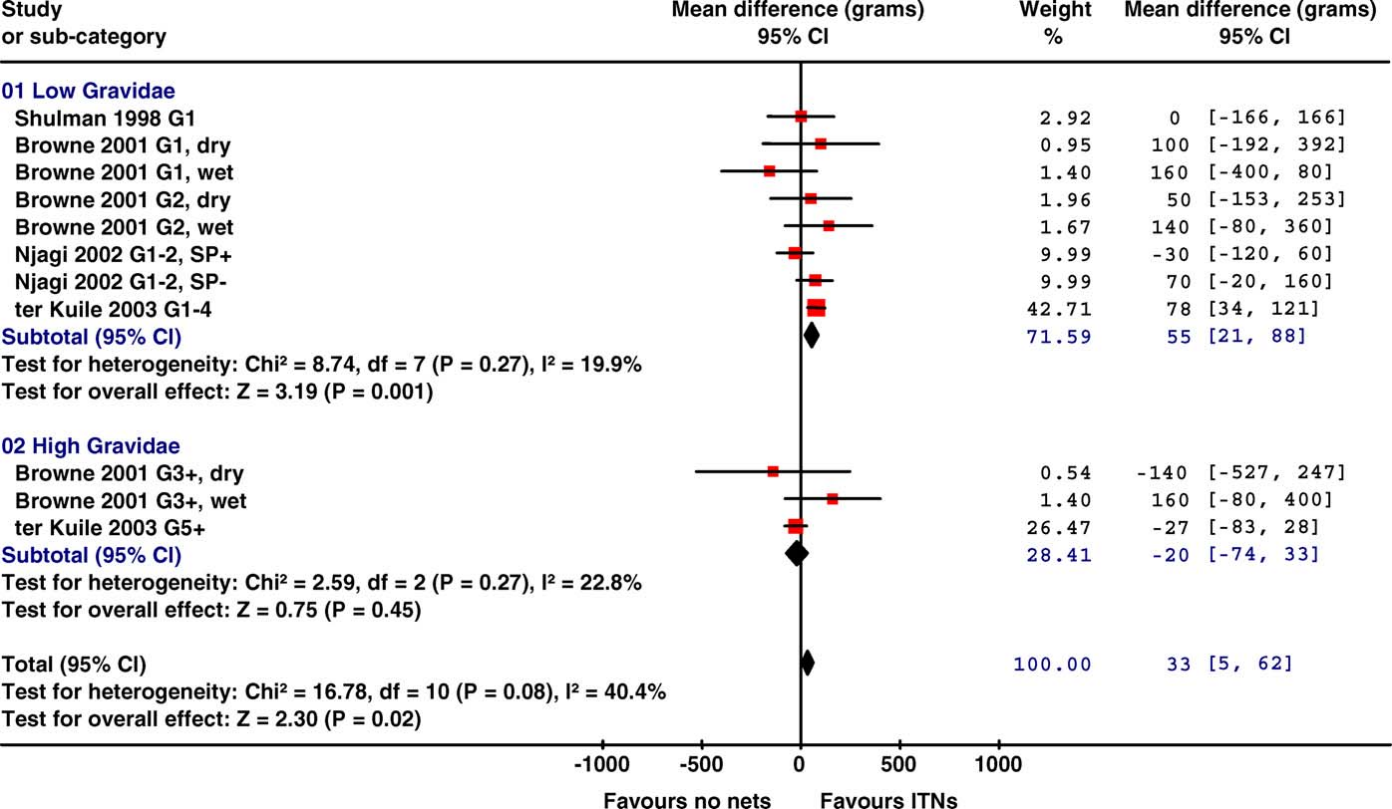
Effect of ITNs versus No Nets in Africa on Mean Birth Weight (in Grams) The red squares represent the effect estimates of ITNs; the black lines represent the 95% confidence intervals associated with the effect estimates. The black diamonds represent the summary effect estimates for the different subgroups (“subtotal”) and for the overall effect (“total”). “Dry” and “wet” refer to the dry and wet seasons. SP+, women randomized to IPTp-SP; SP-, women randomized to receive placebo ITPp (factorial design).

All four trials comparing nets to no nets reported on mean birth weight (Table 2; Figure 2). The average birth weight was 55 g higher in the ITN group in women of low gravidity, but no difference was detected in women of higher gravidity groups. For LBW, two trials contributed (Table 2), indicating women of low gravidity had a 23% reduction in LBW, but there was no apparent effect in women of high gravidity in the one trial measuring this [27]. There was also no evidence for an effect in women receiving IPTp with sulfadoxine-pyrimethamine (IPTp-SP) (one trial) (Figure 2). Browne reported the overall OR adjusted for clustering for all gravidity as 0.87 (95% CI 0.63–1.19); as no information was provided by gravidity group, and because LBW was a common event in this trial, the OR could not be pooled with the relative risk (RR) estimates from the other trials.

**Table 2:**
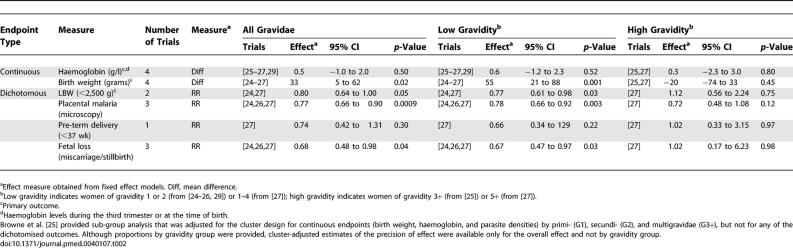
Summary Effect Measures of Four Trials Comparing ITNs versus No Nets in Africa

#### Secondary outcomes.

**Figure 3:**
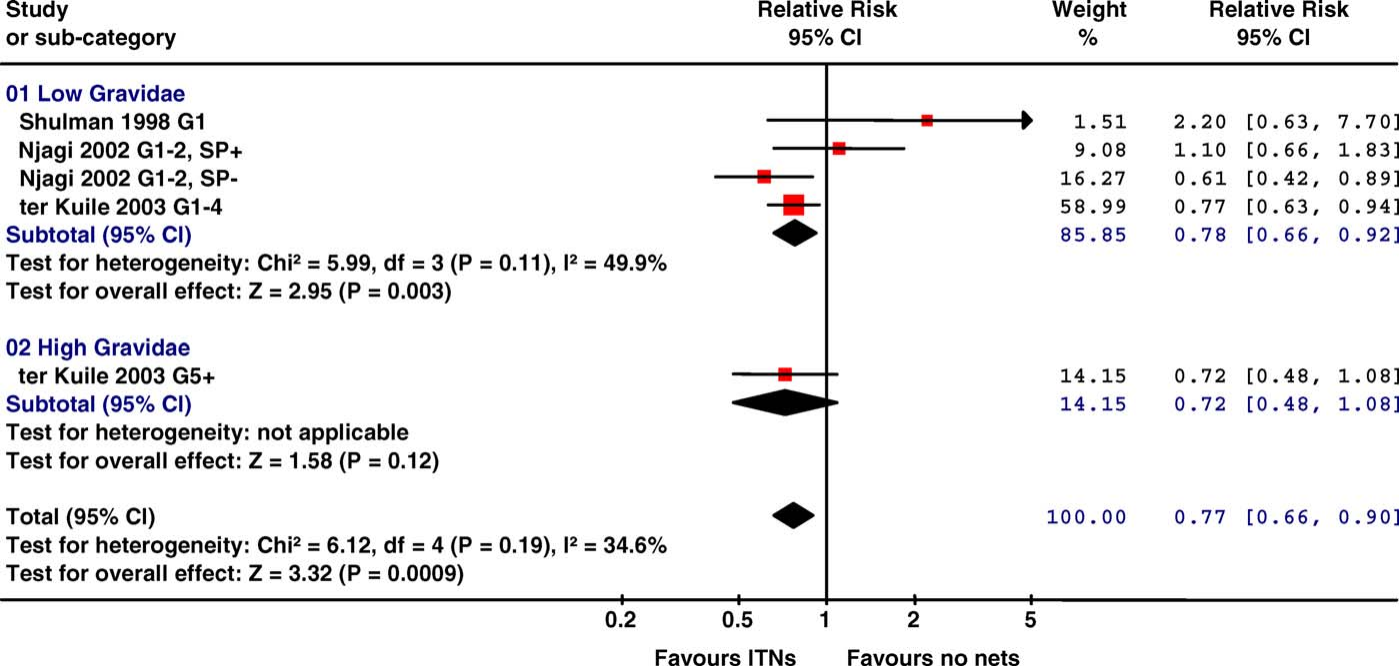
Effect ITNs versus No Nets in Africa on Placental Malaria The red squares represent the effect estimates of ITNs; the black lines represent the 95% confidence intervals associated with the effect estimates (a line with an arrow indicates that the confidence interval was greater than could be illustrated in the graph). The black diamonds represent the summary effect estimates for the different subgroups (“subtotal”) and for the overall effect (“total”). Placental malaria was defined as the presence of asexual parasitaemia detectable by microscopy. Data from Shulman et al. [26] are based on 25.8% of all enrolled women, and includes only women who delivered in the hospital. The degree of heterogeneity approached moderate levels (*I* ^2^ = 49.9%) in the low gravidity group. Similar analysis using random instead of fixed-effect models gave a summary effect of 0.82 (0.61–1.11), 0.72 (0.48–1.08), and 0.79 (0.63–0.98) for low, high, and all gravidae, respectively. SP+, women randomized to IPTp-SP; SP-, women randomized to receive placebo ITPp (factorial design).

All four RCTs reported on malaria parasitaemia. One trial tested women every month and showed time to first infection in the ITN group was reduced (HR 0.67, 95% CI 0.52–0.86, *p =* 0.002) [27]. The prevalence of parasitaemia was less common in the ITN groups when assessed in the third trimester (OR 0.88, 073–1.06, *p =* 0.19, two trials) [25,26] or at the time of delivery (RR 0.76, 0.67–0.86, *p <* 0.001, two trials) [24,27]. Placental malaria parasitaemia was lower with ITNs by 23% (95% CI 10–34, three trials; Table 2). There was no evidence for an effect on the prevalence of peripheral or placental malaria in women who were provided IPTp-SP (one trial, Figure 3) [24].

Geometric mean parasite densities in peripheral blood tended to be lower in the ITN groups in women having their first or second baby, although the result was not statistically significant (geometric mean ratio 0.82, 95% CI 0.66–1.02, *p* = 0.07, two trials) [24,25]. There was no evidence for a beneficial effect in G3+ in the trial by Browne et al. (geometric mean ratio 1.28, 0.90–1.82, *p =* 0.17). Ter Kuile reported that maternal and placental parasite densities were identical in parasitaemic women from ITN and control villages, but insufficient details were provided for inclusion in this analysis [27].

Clinical malaria was reported in two trials, and episodes were less frequent in the ITN than in the control groups in both trials, but this was not significant. Shulman et al. reported on self-reported illness with parasitaemia (OR 0.85, 95% CI 0.47–1.54) [26], and ter Kuile et al. reported on any documented parasitaemia with documented fever based on monthly assessments in G1–G4 (HR 0.72, 95% CI 0.19–2.78) [27].

No effect was demonstrated in the one trial measuring pre-term delivery (<37 wk of gestation) [27] (Table 2).

**Figure 4:**
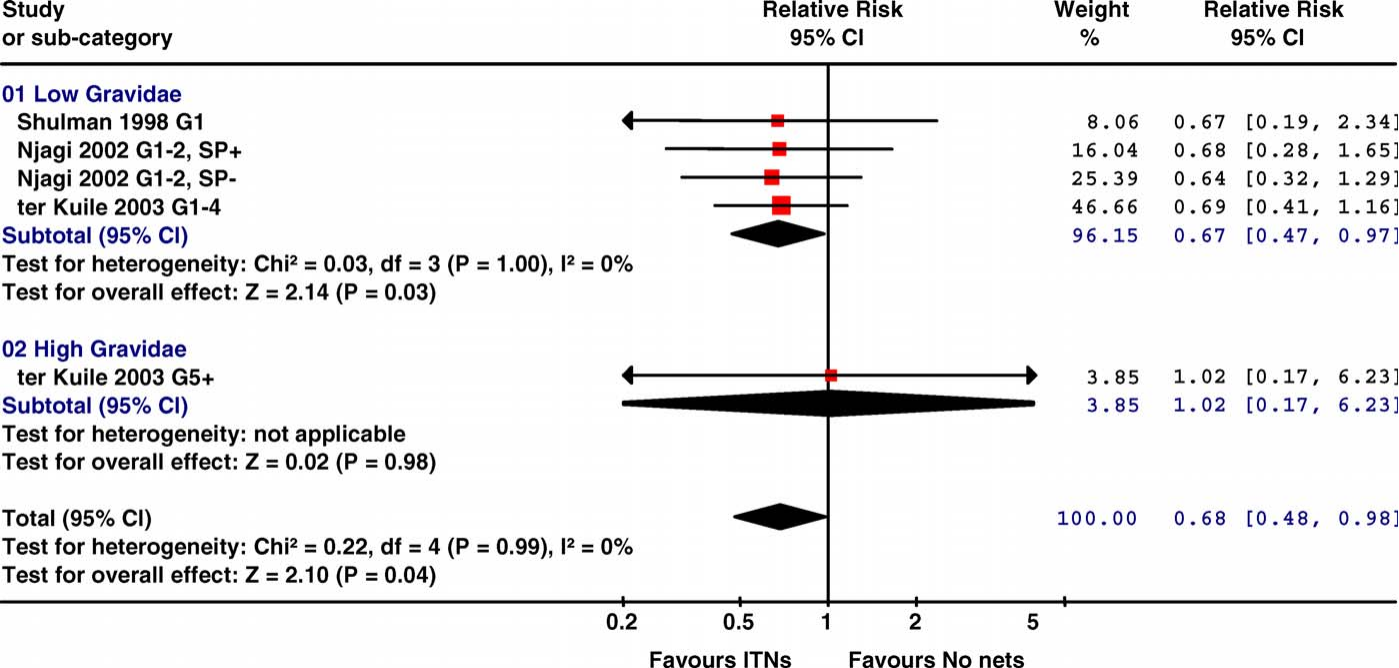
Effect of ITNs versus No Nets in Africa on Miscarriage or Stillbirth The red squares represent the effect estimates of ITNs; the black lines represent the 95% confidence intervals associated with the effect estimates (a line with an arrow indicates that the confidence interval was greater than could be illustrated in the graph). The black diamonds represent the summary effect estimates for the different subgroups (“subtotal”) and for the overall effect (“total”). Data from Shulman et al. [26] refer to stillbirths only. As the event is rare (<10%), the OR reported by Shulman et al. approximates an RR and has been combined with the RRs of Njagi [24] and ter Kuile et al. [27]. SP+, women randomized to IPTp-SP; SP-, women randomized to receive placebo ITPp (factorial design).

The three trials reporting on fetal loss (miscarriage or stillbirth) showed a consistent reduction in fetal loss associated with ITNs in low gravidity women (33%, 95% CI 3–53, *p =* 0.03; Figure 4; Table 2). Browne et al. [25] did not provide a breakdown by intervention group.

Maternal death was reported by Njagi [24] (five deaths), with no trends evident by group; Shulman et al. [26] reported four deaths but did not specify the groups.

### ITNs versus Untreated Nets (One Trial from Thailand)

This trial was conducted on the Thailand–Myanmar border, with individual randomisation [23]. Fewer women experienced peripheral malaria parasitaemia in the ITN group, but this was not significant (RR 0.73, 95% CI 0.47–1.04); however, in women infected with malaria, the geometric mean parasite density was lower in the ITN group (507 versus 1,096, *p =* 0.049), and anaemia (hematocrit < 30%) was less frequent with ITNs (RR 0.63, 95% CI 0.42–0.93). Mean birth weight was similar between the two groups (ITN group, 2,858 g, standard deviation 486, *n =* 94, versus untreated net group, 2,891 g, standard deviation 481, *n =* 85), as was LBW (RR 1.04, 95% CI 0.52–2.07) and pre-term delivery (RR 0.92, 95% CI 0.45–1.88). Fetal loss was significantly lower in the ITN group (2/102, 2%) than the untreated net group (10/97, 10%) (RR 0.21, 95% CI 0.05–0.92). The number of maternal deaths was similar (ITN group, 0/103, versus untreated net group, 2/100).

## Discussion

This systematic review shows that ITNs were associated with some important health benefits for pregnant women and their babies. Women of low gravidity randomised to ITNs delivered fewer LBW babies and were less likely to experience fetal loss (miscarriage or stillbirth). Although the latter was not a primary endpoint in the trials, it is an important outcome. No significant decrease was observed in pre-term deliveries in the single trial that assessed this outcome. The consistent reduction observed in the miscarriage and stillbirth rates suggests that the attributable effect of malaria on fetal loss may be underestimated in malaria-endemic Africa, where most women remain asymptomatic when infected with P. falciparum. Despite the reduction in malaria infections, no overall effect on mean haemoglobin was demonstrated, and data on maternal anaemia were inconsistent.

WHO currently recommends that women in malaria-endemic areas of Africa use both IPTp-SP and ITNs in pregnancy to prevent malaria. One of the two trials from western Kenya assessed the effect of ITNs and IPTp-SP simultaneously, using a factorial design. This trial showed that ITNs provided benefits in primigravidae when used alone, but it did not demonstrate additional benefits of the combined interventions over either of the single interventions [24,29]. The main benefit of ITNs in women protected by IPTp-SP may thus occur after birth through protection of infants from malaria, since infants typically share sleeping space with the mother for the first several months to years [30]. Similar considerations apply to the benefit of ITNs in grand-multigravidae (G5+), as no direct beneficial effect on the developing fetus in terms of birth weight or fetal loss was apparent in this group.

The only trial included in this analysis that compared ITNs to untreated nets was also the only trial conducted outside of Africa, in an area with highly seasonal P. falciparum and P. vivax malaria on the Thailand–Myanmar border. It showed a statistically significant reduction in anaemia and fetal loss in all gravidae, but no evidence for a beneficial effect on birth weight or gestational age [23].

Extrapolation of results from the three cluster-randomised trials to predict the potential impact of programmes that distribute ITNs to individual pregnant women as part of antenatal care should be done with caution. Firstly, nets distributed as part of antenatal care will leave most women exposed to malaria in the first third or half of pregnancy, when the risk of peripheral malaria parasitaemia is greatest [3]. By contrast, most women in the cluster-randomised trials became pregnant after ITNs were distributed and were as such protected throughout pregnancy. Secondly, the effect of ITNs in the cluster-randomised trials reflects the combined effects of personal protection (individual barrier protection) and area-wide reductions in malaria transmission (community or mass effect) [19–21]. It is possible that the mass killing effect on mosquito populations in areas with a high ITN coverage will result in stronger treatment effects of ITNs than can be achieved with individual nets. It is also likely that the community effect in the cluster-randomised trials resulted in a slight underestimation of the magnitude of the effect of ITNs because women living in control households from adjacent villages not using ITNs will have benefited from the area-wide reductions in vector populations, as has been shown for effect estimates in young children [19]. Similar considerations apply to the trial comparing ITNs with untreated nets from the Thailand–Myanmar border [23]. Although, this trial randomised individual women, all trial participants lived in the same densely populated refugee camps and some mass effect by the treated nets cannot be excluded.

The most recent trial from western Kenya by Njagi et al. is informative in this respect, as it is the only trial that compared the effects of ITNs versus no nets using simple randomisation by individual in an area with low ITN coverage (little or no mass effect) [24,29]. This trial and the community-randomised trial by ter Kuile et al. [27] were conducted simultaneously in contiguous areas with similar malaria transmission at baseline, and similar socioeconomic and educational status and ethnicity of the trial population. The effect estimates were similar between the two trials (in women not randomised to IPTp-SP), suggesting that ITNs may work equally well when provided to individuals as part of antenatal care in the second trimester or when provided to entire communities.

The systematic review was informative, but there were some limitations stemming from the variety in trial designs and the number of trials. Outcome data were often expressed in different ways, and inclusion or analysis of gravidity groups was different. How anaemia and peripheral parasitaemia were detected and treated varied, with different periods of follow-up and different cut-offs, limiting our ability to provide summary estimates for some of the endpoints, or to provide sub-group analysis by gravidity group where desired. Shulman et al. and Njagi et al. tested and treated women only if they were suspected of being anaemic or of having malaria, but Dolan et al. performed weekly blood tests, and ter Kuile et al. tested monthly. The number of studies included in the analysis was limited. All four African studies were conducted in areas with stable malaria transmission with EIRs ranging from 10/y to 300/y. Three of the four were conducted in Kenya, and two of these in adjacent areas with similarly intense perennial transmission. These two studies had the greatest influence (expressed as the weight in the figures) on the overall results of the systematic review, particularly for the effect on placental malaria because in the trial by Shulman et al. [26] data were available for only 25.8% of women (those that delivered in the hospital). It is plausible that the 25.8% were different to those delivering at home and may not be representative of all those randomised. This may also explain some of the observed heterogeneity of the effect of ITNs on placental malaria.

Although relatively few trials have been conducted and some questions on the efficacy of ITNs in pregnant women in Africa remain, the four trials comparing ITNs with no nets suggest significant beneficial effects of ITNs on birth weight and fetal loss in the first few pregnancies in areas with moderate to intense malaria transmission in sub-Saharan Africa. These findings are consistent with a non-randomised trial of the effect of socially marketed ITNs conducted in an area with intense perennial malaria transmission in southern Tanzania [31], and with an excluded randomised controlled trial from The Gambia, which has lower and highly seasonal transmission [22]. These observed beneficial effects of ITNs during the first few pregnancies, together with the absence of apparent harm to the developing fetus, the potential beneficial effect on the infant when the net continues to be used after birth [10], and the potential for ITNs to reduce malaria transmission through a mass killing effect on mosquito populations, support the current recommendations from WHO to provide ITNs for pregnant women in all regions with stable malaria transmission throughout sub-Saharan Africa, regardless of the degree of malaria transmission intensity.

Further evaluation of the potential effect of ITNs on pregnant women and their infants is warranted in malaria regions including the Americas, Asia, and the southwest Pacific, which represent approximately half of all pregnant women exposed annually to malaria. The more complex vector populations with exophagic, exophilic, and early biting behaviour in some of these areas may result in lower efficacy of ITNs than in Africa, where *Anopheles gambiae* s.s. is the most important vector. These studies should include women of all gravidae, and ideally address the interaction between ITNs and drug-based prevention such as IPTp, which is also largely untested outside of Africa. In Africa, it took over a decade for the evidence of ITN or IPTp efficacy in pregnant women to accumulate. It would be more efficient if trials had a common design, and if systematic reviews used individual patient data to allow appropriate collection of design effects, more accurate and standardised handling of the data, and more robust sub-group analysis. In order to enhance the rate at which evidence becomes available and is translated into policy, future trials would clearly benefit from better co-ordination between research groups.

## Supporting Information

Text S1 QUOROM Flowchart Screened, excluded, and included number of randomised controlled trials.
(24 KB PPT)

## Abbreviations

CI: confidence interval
EIR: entomological inoculation rate
G[number]: gravidity [number]
Hb: haemoglobin
HR: hazard ratio
IPT: intermittent preventive treatment
IPTp: intermittent preventive treatment in pregnancy
IPTp-SP: intermittent preventive treatment in pregnancy with sulfadoxine-pyrimethamine
ITN: insecticide-treated bednet
LBW: low birth weight
OR: odds ratio
RR: relative risk
SP: sulfadoxine-pyrimethamine
WHO: World Health Organization
