# Supplementary material for: Insecticide-Treated Nets for the Prevention of Malaria in Pregnancy: A Systematic Review of Randomised Controlled Trials
Source: PLoS Med. 2007 Mar 27;4(3):e107. doi: 10.1371/journal.pmed.0040107 (PMC1831739; doi:10.1371/journal.pmed.0040107)
Supplement: Text S1 — Screened, excluded, and included number of randomised controlled trials. (24 KB PPT) [file pmed.0040107.sd001.ppt]

## Slide 1
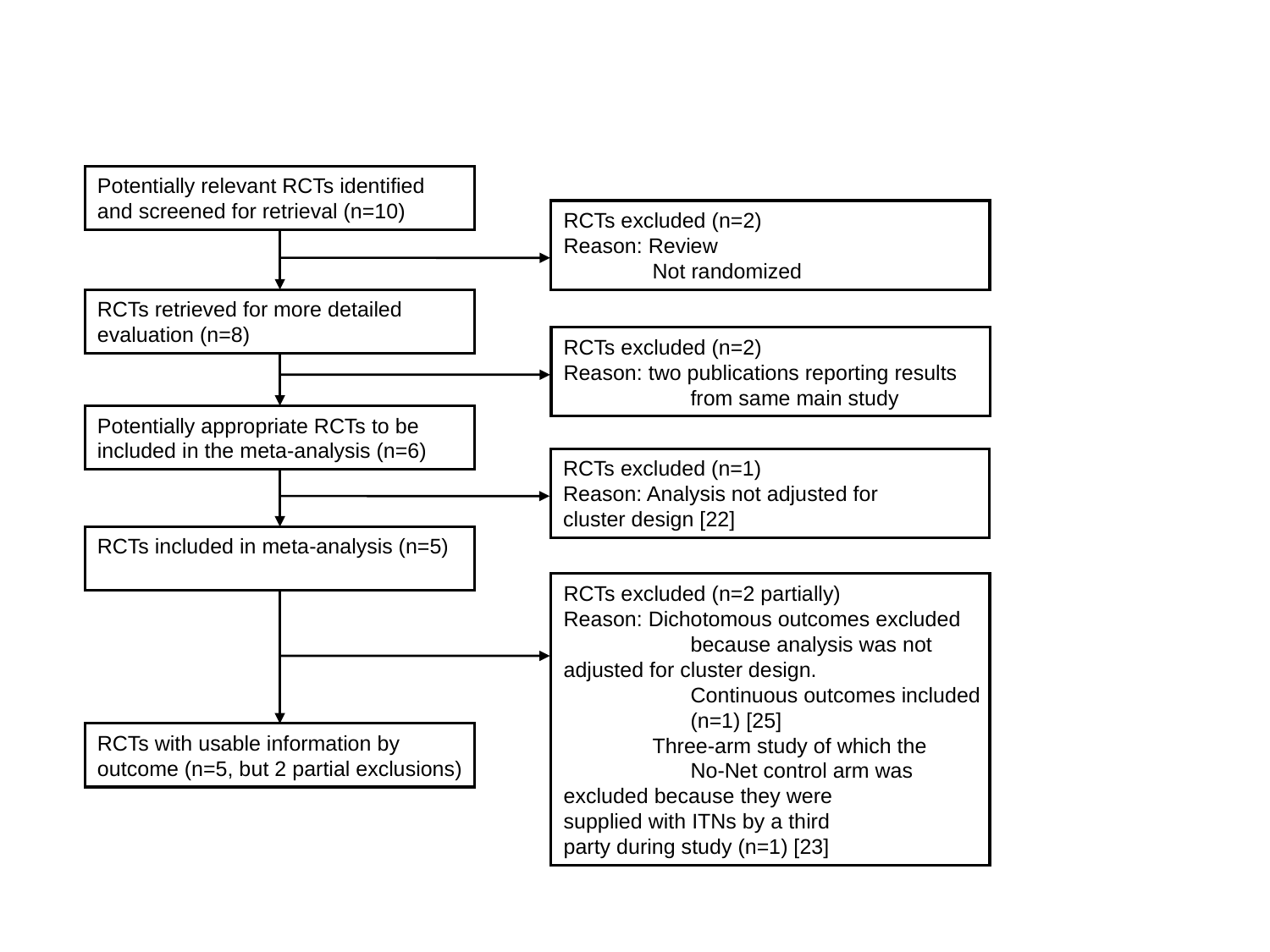

Potentially relevant RCTs identified and screened for retrieval (n=10)
RCTs excluded (n=2)
Reason: Review
 Not randomized
RCTs retrieved for more detailed evaluation (n=8)
RCTs excluded (n=2)
Reason: two publications reporting results 	from same main study
Potentially appropriate RCTs to be included in the meta-analysis (n=6)
RCTs excluded (n=1)
Reason: Analysis not adjusted for 	cluster design [22]
RCTs included in meta-analysis (n=5)
RCTs excluded (n=2 partially)
Reason: Dichotomous outcomes excluded 	because analysis was not 	adjusted for cluster design.		Continuous outcomes included 	(n=1) [25]
 Three-arm study of which the
	No-Net control arm was 	excluded because they were 	supplied with ITNs by a third 	party during study (n=1) [23]
RCTs with usable information by outcome (n=5, but 2 partial exclusions)
